# Mitochondrial DNA Evidence Supports the Hypothesis that *Triodontophorus* Species Belong to Cyathostominae

**DOI:** 10.3389/fmicb.2017.01444

**Published:** 2017-08-03

**Authors:** Yuan Gao, Yan Zhang, Xin Yang, Jian-Hua Qiu, Hong Duan, Wen-Wen Xu, Qiao-Cheng Chang, Chun-Ren Wang

**Affiliations:** ^1^College of Animal Science and Veterinary Medicine, Heilongjiang Bayi Agricultural University Daqing, China; ^2^State Key Laboratory of Agricultural Microbiology, College of Veterinary Medicine, Huazhong Agricultural University Wuhan, China; ^3^College of Life Science and Biotechnology, Heilongjiang Bayi Agricultural University Daqing, China

**Keywords:** complete mitochondrial genome, Strongyloidea, Strongylidae, Cyathostominae, Strongylinae, phylogenetic analysis

## Abstract

Equine strongyles, the significant nematode pathogens of horses, are characterized by high quantities and species abundance, but classification of this group of parasitic nematodes is debated. Mitochondrial (mt) genome DNA data are often used to address classification controversies. Thus, the objectives of this study were to determine the complete mt genomes of three Cyathostominae nematode species (*Cyathostomum catinatum, Cylicostephanus minutus*, and *Poteriostomum imparidentatum*) of horses and reconstruct the phylogenetic relationship of Strongylidae with other nematodes in Strongyloidea to test the hypothesis that *Triodontophorus* spp. belong to Cyathostominae using the mt genomes. The mt genomes of *Cy. catinatum, Cs. minutus*, and *P. imparidentatum* were 13,838, 13,826, and 13,817 bp in length, respectively. Complete mt nucleotide sequence comparison of all Strongylidae nematodes revealed that sequence identity ranged from 77.8 to 91.6%. The mt genome sequences of *Triodontophorus* species had relatively high identity with Cyathostominae nematodes, rather than *Strongylus* species of the same subfamily (Strongylinae). Comparative analyses of mt genome organization for Strongyloidea nematodes sequenced to date revealed that members of this superfamily possess identical gene arrangements. Phylogenetic analyses using mtDNA data indicated that the *Triodontophorus* species clustered with Cyathostominae species instead of *Strongylus* species. The present study first determined the complete mt genome sequences of *Cy. catinatum, Cs. minutus*, and *P. imparidentatum*, which will provide novel genetic markers for further studies of Strongylidae taxonomy, population genetics, and systematics. Importantly, sequence comparison and phylogenetic analyses based on mtDNA sequences supported the hypothesis that *Triodontophorus* belongs to Cyathostominae.

## Introduction

Equine strongyles, a large group of intestinal nematodes that belong to members of Equidae, are classified into two subfamilies, Strongylinae (large strongyles) and Cyathostominae (small strongyles), based on worm size and capsule mouth shape (Lichtenfels et al., 2008; Traversa et al., 2010). Although equine strongyles are the significant nematode pathogens of horses, information on these nematodes is limited to their morphology, prevalence, and disease control and prevention (Bu et al., 2009; Lyons et al., 2011; Mughini et al., 2011; Morariu et al., 2016; Singh et al., 2016). Intestinal nematodes of Cyathostominae, a ubiquitous parasitic nematode species, inhabit the large intestine of infested equines with a high prevalence, especially because of the reduced prevalence of *Strongylus* spp. and spread of cyathostomin anthelmintic-resistant populations (Reinemeyer, 1986; Traversa et al., 2010). Small strongyles can infect virtually any horse, with symptoms of anorexia, weight loss, poor hair coat, lethargy with disordered intestinal motility, and some with inflammatory enteropathy caused by adult Cyathostominae, especially as a result of emergence of enormous numbers of larvae from the lining of the large intestine with a high mortality rate of up to 50% caused by larval stages (Reinemeyer, 1986; Love et al., 1999; Corning, 2009; Traversa et al., 2010).

The traditional classification of equine strongyles was primarily based on morphological characteristics, but some researchers believed that the classification of Strongylidae (including the separation of Strongylinae and Cyathostominae) based on differences in the size and shape of the buccal capsule was arbitrary (Durette-Desset et al., 1994; Lichtenfels et al., 1998). However, reconstruction of the systematic relationships using the first and second internal transcribed spacers (ITS1 and ITS2, respectively) of 30 equine strongyles species revealed that *Triodontophorus serratus*, which was previously classified into Strongylinae based on morphology, clustered with Cyathostominae (Hung et al., 2000).

Mitochondrial (mt) genome sequences provide effective and reliable molecular markers for various types of evolutionary studies of parasites because of their strict maternal inheritance, apparent lack of recombination, rapid evolutionary rate, and comparatively conserved genomic structure (Gissi et al., 2008; Jia et al., 2010; Tian et al., 2015; Zhang et al., 2015; Guo et al., 2016; Liu et al., 2016), especially for classification of higher taxonomic levels. For example, the mt genome provided evidence that *Orientobilharzia turkestanicum* belongs to the genus *Schistosoma*, and is phylogenetically closer to the African schistosome group than to the Asian schistosome group (Wang et al., 2011). In addition, phylogenetic analyses of both nucleotide and amino acid sequence data of mt genome supported the hypothesis that *Dicrocoelium chinensis* and *D. dendriticum* are genetically distinct species (Liu et al., 2014).

Therefore, the objectives of this study were to: (1) determine the complete mt genome sequences of the three nematodes *Cyathostomum catinatum, Cylicostephanus minutus*, and *Poteriostomum imparidentatum*; (2) analyze and compare the mt genomes of Strongylidae nematodes; and (3) reconstruct the phylogenetic relationship of Strongylidae with other nematodes in Strongyloidea based on the mtDNA data to test the hypothesis that *Triodontophorus* belongs to Cyathostominae.

## Materials and methods

### Ethics approval

This study was approved by the Animal Ethics Committee of Heilongjiang Bayi Agricultural University. Horses used for the study were handled in accordance with good animal practice, as defined by the Animal Ethics Procedures and Guidelines of the People's Republic of China.

### Parasites, DNA extraction, genome amplification, and sequence analyses

Adult nematode *Cy. catinatum, Cs. minutus*, and *P. imparidentatum* were collected from the large intestine of naturally infected horses from a slaughter house in Daqing, Heilongjiang Province, China. Individual worms were identified to species based on their morphological characteristics and predilection sites (Lichtenfels et al., 2008). Under the microscope, the morphological characteristics of *Cy. catinatum* including the clear mouth collar, buccal cavity wide in the front and narrow in the back and its width greater than its depth, short dorsal lobe of male bursa and the foot-shaped tail of female could be observed (see Figure S1). *Cs. minutus* is small with a short mouth collar, clear and long submedian papilla, buccal cavity deeper than wide, dorsal gutter elongate reaching half the depth of buccal capsule, short dorsal lobe of male bursa, and straight tails for females (see Figure S2). *P. imparidentatum* are relatively large and have a clear internal and external leaf-crown, high mouth collar, fine and sharp submedian papilla, width of buccal capsule greater than depth, and short and wide dorsal lobe of male bursa (see Figure S3). The detailed data are listed in Table 1.

**Table 1:** The detail information of morphological characteristics of the three nematodes.

	**Female**								**Male**			
	***Cy. c***	**R**	***P. i***	**R**	***Cs. m***	**R**	***Cy. c***	**R**	***Cs. m***	**R**	***P. i***	**R**
Number	3	–	1	–	3	–	3	–	1	–	1	–
Body length/mm	8.9–9.3	4.89–9.8	15.1	11.5–18.4	6.6–6.8	4.6–6.8	7.2–7.5	4.0–8.6	4.8–5.0	4.0–5.2	12.6	11.5–14.0
ELC/elements	22	18–22	86	74–95	8	8	20	18–22	8	8	82	74–95
BC width/μm	48.8–52.3	40–68	232	215–240	24.6–29.6	22–28	45.6–48.8	40–58	20.6–21.9	18–24	196.6	183–256
BC depth/μm	25.3–27.2	20–29	69	66–70	27.3–28.5	26–30	23.2–25.9	19–26	25.0–26.3	24–27	54.2	52–64
Vulva to tail tip/μm	153.3–167	98–195	16,500	16,000–17,900	173.3–182.6	100–187	–	–	–	–	–	–
Anus to tail tip/μm	89.6–93.6	75–110	7,500	6,500–10,000	100–105.3	54–120	–	–	–	–	–	–
Spicule length/mm	–	–	–	–	–	–	1.25–1.30	1.12–1.52	0.596–0.601	0.515–0.672	1.03	0.98–1.11
Dorsal ray length/μm	–	–	–	–	–	–	356–360.6	315–377	150.6–155.2	140–170	427	370–620

*BC, Buccal capsule; R, Reference (Lichtenfels et al., 2008); ELC, external leaf-crown; “–”, no results; Cy. c, Cyathostomum catinatum; P. i, Poteriostomum imparidentatum; Cs. m, Cylicostephanus minutus*.

Subsequently, total genomic DNA was extracted from individual worms using TIANamp Genomic DNA Kit (TIANGEN Biotech, Beijing, China) according to the manufacturer's instructions. Parasite species were determined by PCR amplification of the ITS sequences of *Cy. catinatum, Cs. minutus, P. imparidentatum*, using the universal primers NC5 (5′- GTA GGT GAA CCT GCG GAA GGA TCA TT -3′) and NC2 (5′- TTA GTT TCT TTT CCT CCG CT -3′) reported by Gasser et al. (2008). The ITS sequences of *Cy. catinatum, Cs. minutus*, and *P. imparidentatum* obtained in the present study had 99.8, 99.7, and 99.2% identities to the corresponding available sequences in GenBank (*Cs. minutus*, KM085361.1; *P. imparidentatum*, KP693433.1; *Cy. catinatum*, KF850626.1, respectively), which confirmed the identity of the examined three species. The specific primers for mt genomes of the three worms were designed based on the relatively conserved mt sequences of the Strongylidae horse parasites available in GenBank (Table S1). PCR cycling conditions used to amplify mtDNA of the three nematodes were based on those of a previous report (Gao et al., 2017). After PCR amplification, the positive amplicons were sent to Life Technology Company (Beijing, China) for sequencing. With the aid of bioinformatics software such as MegAlign 5.01, Clustal X 1.83, MEGA 5.0, and tRNAscan-SE 2.0 (http://lowelab.ucsc.edu/tRNAscan-SE/) (Thompson et al., 1997; Burland, 2000; Tamura et al., 2011) and manual analysis, the boundary of each protein-coding gene, transfer RNA (tRNA) gene, and ribosomal RNA (rRNA) gene was determined.

### Comparative analysis with other strongylidae worms

Comparisons were made based on mtDNA size, gene arrangement, percentage of A+T content, and nucleotide and amino acid sequence similarity inferred from individual protein-coding genes among *Cy. catinatum, Cs. minutus, P. imparidentatum*, and nine other Strongylidae nematodes for which mt genome sequences were available in GenBank, which included *Cylicocyclus insigne* (NC_013808.1), *Cs. goldi* (AP017681.1), *Cylicocyclus nassatus* (KX819273.1), *Strongylus equinus* (NC_026868.1), *S. vulgaris* (AP017698.1), *T. brevicauda* (NC_026729.1), *T. nipponicus* (NC_031517.1), *T. serratus* (NC_031516.1), *Macropicola ocydromi* (NC_023099.1).

### Phylogenetic analyses

Phylogenetic analysis in this study was based on the concatenated amino acid sequences of 12 protein-coding genes of 20 Strongyloidea nematodes available in GenBank from family Chabertiidae: *Chabertia erschowi* (KF660603), *C. ovina* (NC_013831); family Cloacinidae: *Oesophagostomum asperum* (NC_023932.1), *O. columbianum* (NC_023933.1), *O. dentatum* (GQ888716), *O. quadrispinulatum* (NC_014181), *Hypodontus macropi* (NC_023099.1); family Strongylidae, including two subfamilies, Cyathostominae (*Cs. goldi, Cc. insigne, Cc. nassatus, Cy. catinatum, Cs. minutus*, and *P. imparidentatum*) and Strongylinae (*S. equinus, S. vulgaris, T. brevicauda, T. nipponicus, T. serratus, M. ocydromi*); and family Syngamidae: *Syngamus trachea* (GQ888718), and family Ancylostomatidae: *Ancylostoma duodenale* (NC_003415) as an outgroup. Each gene was translated using the invertebrate mitochondrial genetic code in MEGA 5 (Tamura et al., 2011) and ambiguously aligned regions were excluded using the Gblocks Server (http://molevol.cmima.csic.es/castresana/Gblocks_server.html) with less stringent selection. Phylogenetic trees were all reconstructed using Bayesian inference (BI) methods, which were performed using the mixed model in MrBayes 3.1.1 and 1,000,000 metropolis-coupled Markov chain Monte Carlo generations (Ronquist and Huelsenbeck, 2003); maximum parsimony (MP) methods, which were performed using a Fitch criterion (1,000 bootstrap replicates) within PAUP 4.0 Beta 10 (Swofford, 2002); and maximum likelihood (ML) methods (JTT+I+G+F model) using PhyML 3.0 (Guindon and Gascuel, 2003), and bootstrapping was performed using 100 replicates. Phylograms were drawn using Tree View 1.65 (Page, 1996).

## Results

### General features of the three MT genomes

In the present study, the complete mt genomes of *Cy. catinatum, Cs. minutus*, and *P. imparidentatum* were 13,838 bp, 13,826 bp and 13,817 bp in length, respectively (Table 2). The mt genomes of *Cy. catinatum* and *P. imparidentatum* are the first reported for both genera. All three mt genomes contained 12 protein-coding genes (*nad*1–*nad*6 and *nad*4L, *cox*1–*cox*3, *cyt*b, *atp*6), 22 tRNA genes, two rRNA genes, and two non-coding regions (Table 2), which were transcribed in the same direction. Moreover, there were 19, 20, and 16 intergenic sequences in the complete circular mt genomes of *Cy. catinatum, Cs. minutus*, and *P. imparidentatum*, respectively. The largest intergenic region occurred in *Cy. catinatum* and was 51 bp in length. The shortest intergenic regions were 1-bp intergenic sequences in all three mt genomes. One, two, and three 1-bp overlaps were present in the mt genomes of *Cy. catinatum, Cs. minutus*, and *P. imparidentatum*, respectively. The total A+T contents in the genome sequences ranged from 74.65 to 76.12%, and the A, T, G, and C contents of the complete mtDNA of *Cy. catinatum, Cs. minutus, P. imparidentatum* ranged from 29.79–30.59%, 44.86–45.53%, 16.66–17.99%, and 7.22–7.37%, respectively. The detailed annotations of the three mt genomes, including the position and length of each gene, A+T content, initiation codon, and termination codon of 12 protein-coding genes, are listed in Table 2.

**Table 2:** Mitochondrial genome organization of three nematodes.

	**Position 5′–3′**			**A**+**T (%)**			**Codons**		
**Genes**	***Cs. m* (13,826 bp)**	***Cy. c* (13,838 bp)**	***P. i* (13,817 bp)**	***Cy. c***	***Cs. m***	***P. i***	**Initiation / termination**		
							***Cy. c***	***Cs. m***	***P. i***
*nad*1	1–873	1–873	1–873	73.88	74.23	72.05	ATT / TAA	TTG/TAG	ATA / TAA
*atp*6	882–1,481	884–1,483	882–1,481	75.15	77.83	74.50	ATT / TAA	ATT/TAA	ATT / TAA
tRNA-Lys (K)	1,511–1,573	1,497–1,559	1,488–1,550						
tRNA-Leu^UUR^ (L2)	1,588–1,642	1,581–1,635	1,570–1,624						
tRNA-Ser^AGN^ (S1)	1,643–1,695	1,636–1,688	1,625–1,677						
*nad*2	1,696–2,541	1,689–2,534	1,678–2,523	79.43	79.91	78.72	TTG / TAA	TTG/TAA	ATT / TAA
tRNA-Ile(I)	2,559–2,618	2,542–2,600	2,526–2,584						
tRNA-Arg (R)	2,620–2,674	2,615–2,699	2,606–2,660						
tRNA-Gln (Q)	2,689–2,743	2,676–2,730	2,678–2,632						
tRNA-Phe (F)	2,746–2,808	2,732–2,787	2,733–2,788						
*cyt*b	2,803–3,915	2,788–3,900	2,789–3,901	71.61	72.51	69.63	ATT / TAA	ATT/TAA	ATA / TAA
tRNA-Leu^CUN^ (L1)	3,922–3,976	3,917–3,971	3,922–3,975						
*cox*3	3,977–4,742	3,972–4,737	3,976–4,741	71.93	72.06	70.10	ATT / T	ATT/T	ATT / T
tRNA-Thr (T)	4,743–4,800	4,738–4,792	4,742–4,797						
*nad*4	4,801–6,030	4,793–6,022	4,798–6,024	76.99	78.05	76.20	TTG / TAA	TTG/TAA	TTG / TAA
SNCR	6,031–6,115	6,023–6,112	6,025–6,108	82.22	82.35	88.10			
*cox*1	6,116–7,693	6,113–7,690	6,109–7,686	69.58	69.39	68.69	ATT / TAA	ATT/TAA	ATT / TAA
tRNA-Cys (C)	7,693–7,749	7,690–7,744	7,686–7,740						
tRNA-Met (M)	7,764–7,822	7,755–7,813	7,741–7,800						
tRNA-Asp (D)	7,826–7,884	7,819–7,877	7,806–7,867						
tRNA-Gly (G)	7,900–7,955	7,889–7,944	7,884–7,939						
*cox*2	7,957–8,652	7,945–8,640	7,940–8,635	71.55	73.56	71.41	ATT / TAA	ATT/TAA	ATA / TAA
tRNA-His (H)	8,656–8,709	8,646–8,699	8,635–8,688						
*rrn*L	8,710–9,681	8,700–9,675	8,689–9,671	80.84	81.60	81.28			
*nad*3	9,682–10,017	9,676–10,011	9,672–10,007	76.19	78.57	75.89	ATT / TAG	ATT/TAA	ATT / TAA
*nad*5	10,033–11,616	10,029–11,612	10,024–11,607	77.27		76.64	ATT / TAA	ATT/TAA	ATT / TAG
tRNA-Ala (A)	11,620–11,675	11,616–11,671	11,607–11,662						
LNCR	11,676–11,937	11,672–11,942	11,663–11,950	86.72	87.02	83.33			
tRNA-Pro (P)	11,938–11,993	11,943–11,997	11,951–12,005						
tRNA-Val (V)	12,032–12,086	12,009–12,062	12,038–12,091						
*nad*6	12,087–12,521	12,063–12,497	12,092–12,526	79.77	81.15	77.47	ATT / TAG	ATT/TAA	ATG / TAA
*nad*4L	12,560–12,793	12,548–12,781	12,558–12,791	79.06	79.91	79.91	ATT / TAA	ATT/TAA	ATT / TAA
tRNA-Trp (W)	12,815–12,872	12,801–12,857	12,816–12,872						
tRNA-Glu (E)	12,899–12,957	12,899–12,955	12,875–12,931						
*rrn*S	12,958–13,657	12,956–13,663	12,932–13,640	77.26	78.57	70.10			
tRNA-Ser^UCN^ (S2)	13,658–13,714	13,664–13,719	13,641–13,693						
tRNA-Asn (N)	13,714–13,768	13,720–13,776	13,695–13,750						
tRNA-Tyr (Y)	13,772–13,826	13,781–13,838	13,763–13,817						

*Cy. c, Cyathostomum catinatum; P. i, Poteriostomum imparidentatum; Cs. m, Cylicostephanus minutus*.

### Comparative analysis

Homology analysis showed that the highest identity (91.6%) occurred between the mt genome of *Cy. catinatum* and that of *Cs. goldi* (Table 3). Compared with the three nematodes of *Strongylus* (*S. equinus* and *S. vulgaris*) and *Macropicola* (*M. ocydromi*) in the same subfamily (Strongylinae), the worms in Cyathostominae showed relatively higher similarities to *Triodontophorus* species. Furthermore, the results from these analyses indicate that the mt genome arrangements of the Strongyloidea nematodes sequenced to date are identical. The detailed comparison is listed in Tables 2–4.

**Table 3:** The comparative analysis of mt DNA among the family Strongylidae.

**Subfamily**	**Species**	***Cy. c***	***P. i***	***Cs. m***	***Cs. g***	***Cc. n***	***Cc. i***	***S. e***	***S. v***	***T. b***	***T. n***	***T. s***	***M.o***
Cyathostominae	*Cy. c*	100	–	–	–	–	–	–	–	–	–	–	–
	*P. i*	84.7	100	–	–	–	–	–	–	–	–	–	–
	*Cs. m*	87.5	84.2	100	–	–	–	–	–	–	–	–	–
	*Cs. g*	91.6	85.2	87.6	100	–	–	–	–	–	–	–	–
	*Cc. n*	87.9	84.4	86.7	87.7	100	–	–	–	–	–	–	–
	*Cc. i*	88.2	84.5	87.3	88.4	89.3	100	–	–	–	–	–	–
Strongylinae	*S. e*	79.2	77.8	78.9	79.1	79.0	79.3	100	–	–	–	–	–
	*S. v*	81.0	79.4	80.4	81.1	80.5	80.9	78.7	100	–	–	–	–
	*T. b*	82.9	81.4	82.8	83.0	82.3	83.3	78.8	79.2	100	–	–	–
	*T. n*	85.0	83.6	84.7	85.2	83.9	84.9	78.2	80.3	83.3	100	–	–
	*T. s*	84.6	83.7	84.7	84.9	83.9	84.7	78.8	80.7	84.0	85.6	100	–
	*M.o*	81.1	79.9	80.9	81.3	80.9	81.3	77.1	80.7	78.2	81.0	81.1	100

*Cy. c, Cyathostomum catinatum; P. i, Poteriostomum imparidentatum; Cs. m, Cylicostephanus minutus; Cc. n, Cylicocyclus nassatus; Cc. i, Cylicocyclus insigne; Cs. g, Cylicostephanus goldi; S. e, Strongylus equinus; S. v, Strongylus vulgaris; T. b, Triodontophorus brevicauda; T. n, Triodontophorus nipponicus; T. s, Triodontophorus serratus; M. o, Macropicola ocydromi*.

**Table 4:** The complete nucleotide identify analyses of 12 nematodes in the family Strongylidae.

**Genes**	**No. aa**													**No. nt (bp)**												
	***Cy. c***	***Cs. m***	***P. i***	***Cc.n***	***Cc. i***	***Cs. g***	***S. e***	***S. v***	***T. b***	***T. n***	***T. s***	***M. o***	**aa s (%)**	***Cy. c***	***Cs. m***	***P. i***.	***Cc.n***	***Cc. i***	***Cs. g***	***S. e***	***S. v***	***T. b***	***T. n***	***T. s***	***M.o***	**nt s (%)**
*cox*1	525	525	525	525	525	525	525	525	525	525	525	523	90.1–99.0	1,578	1,578	1,578	1,578	1,578	1,578	1,578	1,578	1,578	1,578	1,578	1,572	83.6–89.9
*cox*2	231	231	231	231	231	231	231	231	231	231	231	231	94.4–99.6	696	696	696	696	696	696	696	696	696	696	696	696	83.5–91.1
*rrn*L														976	973	983	974	959	972	959	959	975	976	961	960	
*nad*3	111	111	111	111	111	111	111	111	111	111	111	112	80.2–98.2	336	336	336	336	336	336	336	336	336	336	336	339	78.6–90.8
NCR3																									107	
*nad*5	527	527	527	527	527	530	532	527	527	527	527	520	80.0–97.3	1,584	1,584	1,584	1,584	1,584	1,593	1,599	1,584	1,584	1,584	1,584	1,561	80.1–90.2
LNCR														271	262	288	270	274	272	271	383	336	259	278	230	
NCR3																				192	89					
*nad*6	144	144	144	144	144	144	144	144	144	144	144	142	77.8–98.6	435	435	435	435	435	435	435	435	435	435	435	429	77.5–89.4
*nad*4L	77	77	77	77	77	77	77	77	77	77	77	77	79.2–98.7	234	234	234	234	234	234	234	234	234	234	234	234	82.9–93.2
NCR3																						166				
*rrn*S														708	700	709	699	700	699	708	700	703	696	700	701	
*nad*1	290	290	290	290	290	290	292	291	290	290	290	284	79.7–99.0	873	873	873	873	873	873	879	876	873	873	873	855	79.5–91.1
*atp*6	199	199	199	199	199	199	199	200	199	199	199	199	84.9–99.0	600	600	600	600	600	600	600	603	600	600	600	600	82.0–91.8
*nad*2	281	281	281	281	281	281	281	281	281	281	281	277	77.2–97.2	846	846	846	846	846	846	846	846	846	846	846	834	77.9–91.5
*cyt*b	370	370	370	370	370	370	370	371	370	370	370	370	80.3–97.8	1,113	1,113	1,113	1,113	1,113	1,113	1,113	1,116	1,113	1,113	1,113	1,113	79.1–90.9
*cox*3	255	255	255	255	256	255	255	255	253	255	255	255	92.2–98.0	766	766	766	766	769	766	766	766	760	766	766	766	82.8–90.5
*nad*4	409	409	408	409	409	409	409	409	408	409	409	401	82.0–98.5	1,230	1,230	1,227	1,230	1,230	1,230	1,230	1,230	1,227	1,230	1,230	1,206	82.0–92.2
SNCR														90	85	84	79	88	87	96	126	89	89	109	75	
Total AA	3,419	3,419	3,418	3,419	3,420	3,422	3,426	3,422	3,416	3,419	3,419	3,391														
Total size (bp)														13,838	13,826	13,817	13,846	13,828	13,827	14,545	14,301	14,305	13,701	13,794	13,659	78.2–91.6
A+T (%)														76.12	76.77	74.65	74.74	76.58	76.08	78.10	76.55	77.15	76.05	77.21	75.82	

Cy. c, Cyathostomum catinatum; P. i, Poteriostomum imparidentatum; Cs. m, Cylicostephanus minutus; Cc. n, Cylicocyclus nassatus; Cc. i, Cylicocyclus insigne; Cs. g, Cylicostephanus goldi; S. e, Strongylus equinus; S. v, Strongylus vulgaris; T. b, Triodontophorus brevicauda; T. n, Triodontophorus nipponicus; T. s, Triodontophorus serratus; M. o, Macropicola ocydromi; nt s, nucleotides similarity; aa s, Amino acid similarity

Comparison of the 12 protein-coding genes of all Strongylidae nematodes revealed that the *nad*4L gene is the most conserved gene in terms of nucleotide sequences; however, the *cox*2 gene is the most conserved at the amino acid level (Table 4). For the codon usage of the 12 protein-coding genes of the three worms analyzed in this study, ATT initiation codons were present in high frequency, TAA was the most shared termination codon, and incomplete codon T appeared at the end of the *cox*3 gene of the three nematodes.

### Phylogenetic analysis

**Figure 1:**
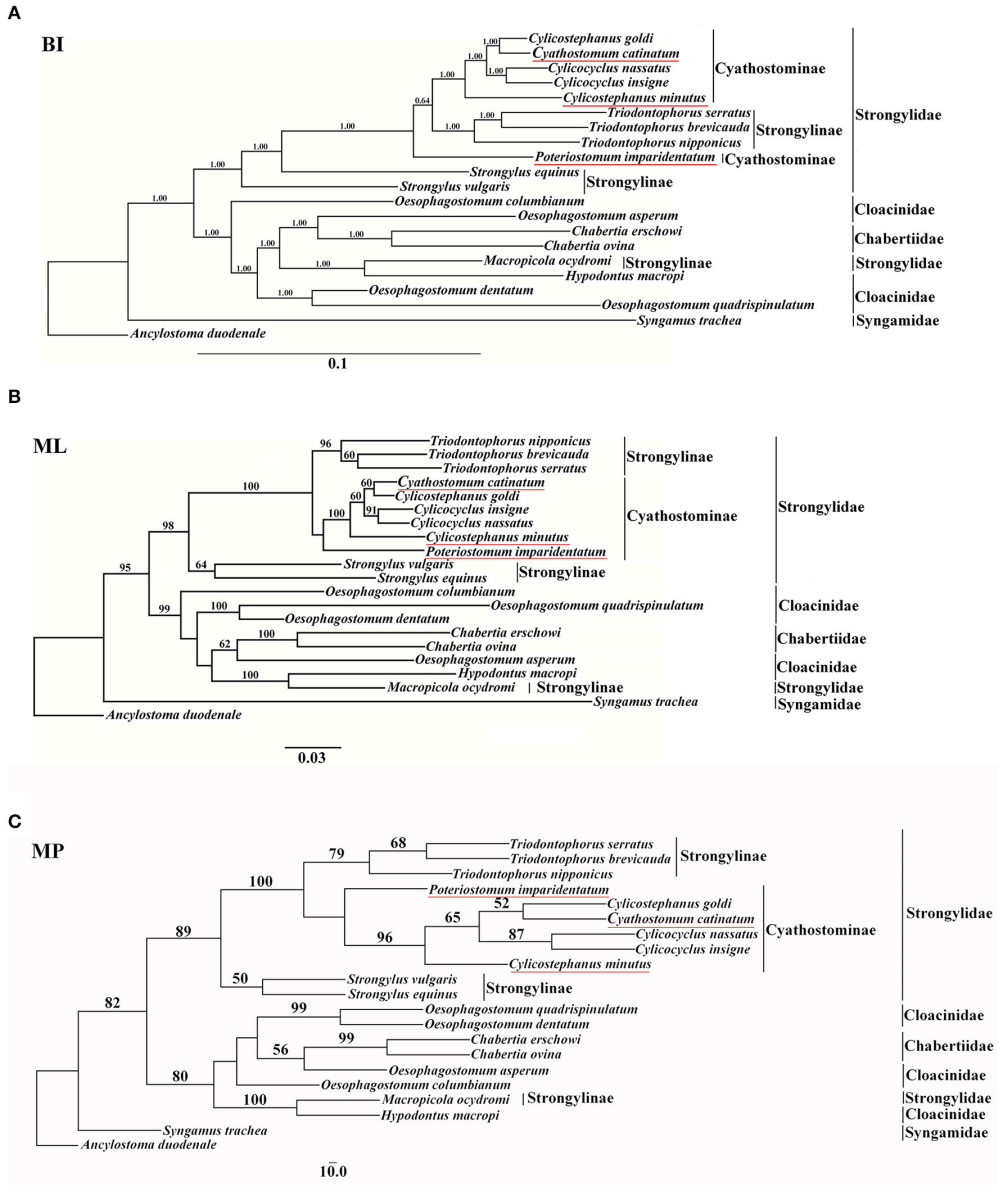
Phylogenetic relationships of 20 Strongyloidea nematodes based on concatenated amino acid sequences of 12 protein-coding genes were analyzed by Bayesian inference (BI) **(A)**, maximum parsimony (MP) **(C)**, and maximum likelihood (ML) **(B)** using *Ancylostoma duodenale* as an outgroup. The values below 50% are not shown.

In the present study, mtDNA data were employed to assess the phylogenetic relationships of Strongyloidea nematodes, and the results are presented in Figure 1. All phylogenetic trees generated similar topologies, and the horse-parasitizing nematodes always clustered together in each tree. However, phylogenetic analysis using different methods revealed different relative positions for some species, such as *P. imparidentatum*. MP and ML trees showed the same topology, except for the branch that contained *O. columbianum* (Figures 1B,C). Congeneric species always formed clades (*T. brevicauda, T. nipponicus*, and *T. serratus*; *Cc. insigne* and *Cc. nassatus*; and *Ch. erschowi* and *Ch. ovina*), and the genera *Oesophagostomum* and *Cylicostephanus* are polyphyletic. *Cs. goldi* and *Cy. catinatum* formed sister taxa in all three trees. *P. imparidentatum* was sister to a clade composed of *Cc. insigne, Cs. goldi, Cc. nassatus, Cy. catinatum, T. brevicauda, T. nipponicus*, and *T. serratus* in the BI tree. However, *P. imparidentatum* clustered with all Cyathostominae nematodes in the MP and ML trees. *Triodontophorus* species clustered together with all the Cyathostominae nematodes with strong support in the three phylogenetic trees. In particular, in the BI tree, the *Triodontophorus* species clustered with *Cylicostephanus, Cyathostomum, Cylicocyclus*, and *Poteriostomum* within the clade of Cyathostominae. The two *Strongylus* species formed sister groups in the ML and MP trees, but not in the BI tree. The kangaroo-parasitizing nematodes (*H. macropi* and *M. ocydromi*) formed a distinct branch with the worms of cattle, sheep, goats, and pigs (*C. erschowi, C. ovina, O. asperum, O. columbianum, O. dentatum, O. quadrispinulatum*), even though *M. ocydromi* is classified in Strongylinae. The *Oesophagostomum* species did not form a monophyletic group, because of *H. macropi, M. ocydromi*, and the *Chabertia* species. However, *O. quadrispinulatum* and *O. dentatum* formed a distinct branch, and *O. asperum* was sister to the *Chabertia* species in all three trees. *Syngamus trachea* was divergent from all other species in the three analyses (see Figure 1).

## Discussion

The mitochondrial genome is important in molecular biology, and is extensively applied when studying the taxonomy, population genetics, and systematics of parasites (Gissi et al., 2008; Zhang et al., 2015; Guo et al., 2016; Liu et al., 2016). In the present study, we obtained the mtDNA of three Cyathostominae nematodes for the first time and compared these mtDNA sequences those of Strongylidae species. The complete mtDNA sequences of the three nematodes were slightly different in length compared with the other Strongylidae nematodes (except for *S. equinus* and *T. brevicauda*), which is consistent with the length of typical metazoan mt genomes (Gissi et al., 2008; Jia et al., 2010; Tian et al., 2015; Zhang et al., 2015; Guo et al., 2016; Liu et al., 2016). Because of the longer length of the non-coding regions, the *S. equinus* and *T. brevicauda* mt genomes were slightly longer than other Strongylidae nematodes mt genomes. This phenomenon is also found in the mt genomes of entomopathogenic nematodes and plant-parasitic nematodes. For example, the lengths of the complete mt genomes of the entomopathogenic nematode *Steinernema litorale* and the plant-parasitic nematode *Pratylenchus vulnus* are 21,403 bp and 21,656 bp in size, respectively; however, the size of the non-coding regions was extremely long (*S. litorale*, 8,137 bp; *P. vulnus*, 7,748 bp) (Sultana et al., 2013; Taisei et al., 2016). The total A+T contents of three worms mtDNA of this study were consistent with those of most nematode mt genomes characterized to date, such as *T. brevicauda* (77.0%) and *Wuchereria bancrofti* (74.6%) (Ramesh et al., 2012; Duan et al., 2015), whereas the percentages of A+T content are remarkably higher than those of trematodes and protozoa, such as *Echinostoma hortense* (63.03%) and *Eimeria magna* (65.16%) (Tian et al., 2015; Liu et al., 2016). The A+T content in non-coding regions was higher than those in other regions of the mt genomes, which is consistent with the mt genomes of other nematodes in previous studies (Hu et al., 2002; Lin et al., 2012; Ramesh et al., 2012). However, non-coding regions quantity differed from those of other nematodes in the same family, and the third non-coding region was found in the mt genomes of *T. brevicauda, Strongylus equinus*, and *M. ocydromi*. There are also several pairs of repeats in the long non-coding regions of the three nematodes in this study with 12 bp for direct repeats and 12–14 bp for inverted repeats. This phenomenon was also found in the long non-coding regions of other helminthes, such as *Strongyloides stercoralis, W. bancrofti, Fascioloides magna, Taenia multiceps, T. hydatigena*, and *T. pisiformis*, but the lengths of those repeat sequences were longer than those in the three worms in the present study (Hu et al., 2003; Jia et al., 2010; Ramesh et al., 2012; Ma et al., 2016). Nevertheless, the functions of these regions remain unclear.

The mt genome of the three nematodes was encoded on the same strand and transcribed in the same direction, which is consistent with those of other Chromadorea nematodes available in GenBank, but different from those of Enoplea nematodes, such as *Trichinella spiralis, Trichuris trichiura*, and *T. ovis* (Lavrov and Brown, 2001; Liu et al., 2012a,b). Interestingly, the gene order in this study is also identical to that of 17 species that belong to superfamily Strongyloidea, for which mt genomes are available in GenBank, including Strongylidae (*Cs. goldi, Cc. insigne, Cc. nassatus, S. equinus, S. vulgaris, T. brevicauda, T. nipponicus, T. serratus*, and *M. ocydromi*), Chabertiidae (*C. erschowi* and *C. ovina*), Cloacinidae (*O. asperum, O. columbianum, O. dentatum, O. quadrispinulatum*, and *H. macropi*), and Syngamidae (*Syngamus trachea*). This phenomenon was also found in another group of the family Trichuridae, including *Trichuris trichiura, T. suis, T. ovis*, and *T. discolor*, whose mt genomes have the same gene arrangement. However, in other nematodes, such as the family Oxyuridae, *Syphacia obvelata* mt genome gene arrangement is consistent with that of *Wellcomia siamensis*, but different from those of *Enterobius vermicularis* and *Aspiculuris tetraptera* (Wang et al., 2016).

The results of the present study showed that topologies of the three trees with different phylogenetic reconstruction strategies were identical or similar. The paraphyly of *Oesophagostomum* species and *Cylicostephanus* species caused by *Chabertia* species and *Cy. catinatum* was evident in the mtDNA analyses. These paraphyletic groups were also proposed by some previous studies based on rDNA sequence (Hung et al., 2000; Gouý de Bellocq et al., 2001). *P. imparidentatum* was evolutionarily distant from other Cyathostominae and *Triodontophorus* species. Additionally, *P. imparidentatum* displayed the lowest nucleotide identity when compared with the other nematodes (*Cy. catinatum, Cc. nassatus, Cc. insigne, Cs. minutus, Cs. goldi, T. brevicauda, T. nipponicus*, and *T. serratus*). The phylogenetic analysis indicated that subfamily Cyathostominae was closer to the *Triodontophorus* species that belong to Strongylinae than to the *Strongylus* species that belong to Strongylinae.

This phenomenon was also reflected in the complete nucleotide identity comparisons; the *Strongylus* species showed relatively low nucleotide identity to the sequences of other equine strongyles. Nevertheless, the *Triodontophorus* worms mtDNA sequences have higher identity with the Cyathostominae than that of *Strongylus* species, which is consistent with the phylogenetic results. The similarity results were also further supported by other studies. The mtDNA-based phylogenetic trees revealed that all the worms in Cyathostominae, *T. serratus*, and *Craterostomum acuticaudatum* clustered with each other rather than the three other nematodes of Strongylinae (*S. equinus, S. vulgaris, S. edentatus*) (McDonnell et al., 2000). Another phylogenetic analysis of the large subunit rDNA D3 domain for 21 species of equine strongyles showed that *T. serratus* and *T. brevicauda* clustered with Cyathostominae rather than Strongylinae (Zhang et al., 2007). Furthermore, there is also biological evidence that supports this relationship; for example, a comparative study of the morphology of the L4s of 20 equine strongyles revealed that the larvae of *Triodontophorus* were more similar to those of Cyathostominae than to those of *Strongylus* species (Dvojnos and Kharchenko, 1990).

The phylogenetic relationships based on the complete mt genomes in this study indicated that the relationships among equine strongyles are inconsistent with the traditional classification. *Triodontophorus* species are genetically different from *Strongylus* species, and are more closely related to Cyathostominae. These analyses supported our hypothesis that *Triodontophorus* species belong to Cyathostominae.

## Conclusions

The findings of the present study, which used comparative and phylogenetic analyses of mtDNA sequences, supported the hypothesis that *Triodontophorus* species belong to Cyathostominae. The availability of the complete mt genome sequences of *Cy. catinatum, Cs. minutus*, and *P. imparidentatum* can provide novel genetic markers for further studies on the taxonomy, population genetics, and systematics of Strongylidae nematodes.

## Data availability statement

Representative nucleotide sequences were deposited in GenBank with the following accession numbers: KY495600-KY495602 for the mtDNA, and KY495603-KY495605 for the ITS.

## Author contributions

CRW conceived and designed the study, and critically revised the manuscript. YG, HD, and WWX performed the experiments. YG and QCC analyzed the data. YG drafted the manuscript. YZ, XY, and JHQ helped in study design, study implementation and manuscript preparation. All authors read and approved the final manuscript.

### Conflict of interest statement

The authors declare that the research was conducted in the absence of any commercial or financial relationships that could be construed as a potential conflict of interest.

## Supplementary material

Table S1 The primers used for obtain mitochondrial genomes of the three nematodes.

Figure S1 The detail information of morphological characteristics of *Cyathostomum catinatum*. **(A)** The head of *Cy. catinatum*, **(B)** The tail of female, **(C)** The tail of male, Bar = 0.1 mm.

Figure S2 The detail information of morphological characteristics of *Cylicostephanus minutus*. **(A)** The head of *Cs. minutus*, **(B)** The tail of female, **(C)** The tail of male, Bar = 0.1 mm.

Figure S3 The detail information of morphological characteristics of *Poteriostomum imparidentatum*. **(A)** The head of *P. imparidentatum*, **(B)** The tail of female, **(C)** The tail of male, Bar = 0.1 mm.
