# Supplementary material for: Mitochondrial DNA Evidence Supports the Hypothesis that Triodontophorus Species Belong to Cyathostominae
Source: Front Microbiol. 2017 Aug 3;8:1444. doi: 10.3389/fmicb.2017.01444 (PMC5540935; doi:10.3389/fmicb.2017.01444)
Supplement: Table S1 — The primers used for obtain mitochondrial genomes of the three nematodes. [file Table1.PDF]

TABLE S1 | The primers used for obtain mitochondrial genomes of the three nematodes

Name of primer	Primer sequence (5'- 3')	
	Cylicostephanus minutus	***Cyathostomum catinatum / Poteriostomum imparidentatum***
*cox*1-*cox*2	F: AATTGGTGGATTTGGTAATTGAA	-
	R: AAAGTTGGGAACACACTACATAACA	-
*cox*2-*rrn*L	F: TATTTGAGTATGTTTGATTTCC	-
	R: TCTTAAAAATACTTCAACTTATACTTT	-
*cox*2-*nad*3	F: AGCTAATCATAGTTTTATGCCTATTGC	-
	R: AAAGAATTTTGAATTTTACCTACCCTT	-
*nad*3-*nad*5	F: GGGAGAAGATTTAATTTTTGAGAC	-
	R: ACCACACAAACAAAACAAAGT	-
*nad*5-*nad*6	F: AAGCTATAAGAGCACCCACTCCT	-
	R: TTTGATAATCTGGAAAAATAAACCAA	-
*nad*6-*rrn*S	F: TTTAAGTATGCTAATGTGTATGCCT	F:TGGGTGTATCGTTATTAAGAGGAGT
	R: GCAATAGCCTACAAAATAAAAACC	R:GTTCCCCTAAATCTACTTTACTAC
*rrn*S-*atp6*	F: AAAGGGGAGTAAGTTGTAGTAAAGTAG	-
	R: ATTCCACAAGGACAAAAAGAATA	-
*nad*1-*cyt*b	F: TTATTGCGGAGTTAAATCGT	-
	R: AGCTCATACTAATACATAGCCCATA	-
*cyt*b-*nad*4	F: TTGGTTGGATTTTTCGTATTTTTC	-
	R: TAGGAGCCTCTACATGAGCTTTTG	-
*nad*4-*cox*1	F: GGTTATGGTTCTCAAATTG	-
	R: CTAATAATACCAAAAGCAGG	-

Note: “-” represents the primer same as the primer of *Cylicostephanus minutus.*
