# Supplementary figures and images for: Mitochondrial DNA Evidence Supports the Hypothesis that Triodontophorus Species Belong to Cyathostominae
Source: Front Microbiol. 2017 Aug 3;8:1444. doi: 10.3389/fmicb.2017.01444 (PMC5540935; doi:10.3389/fmicb.2017.01444)

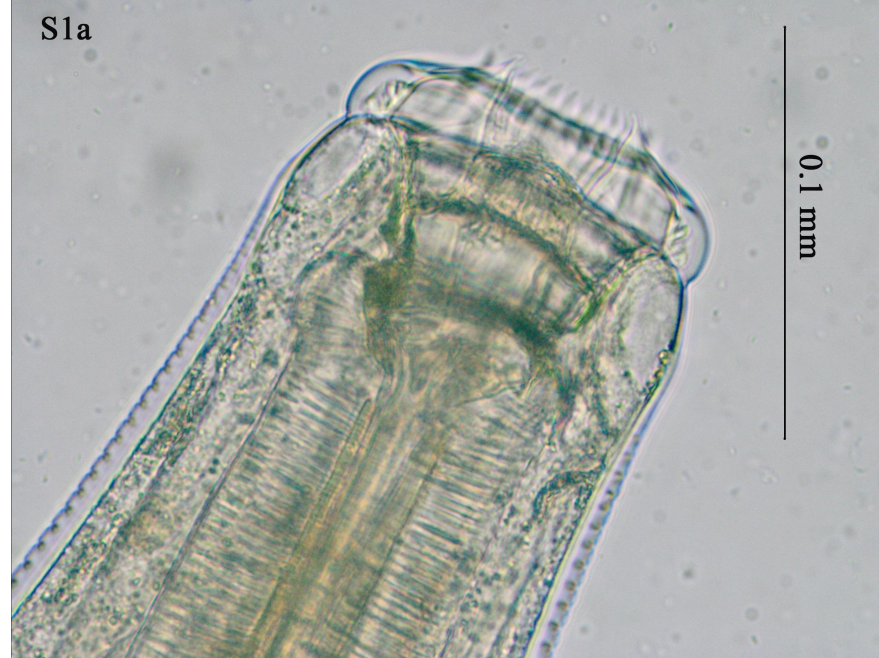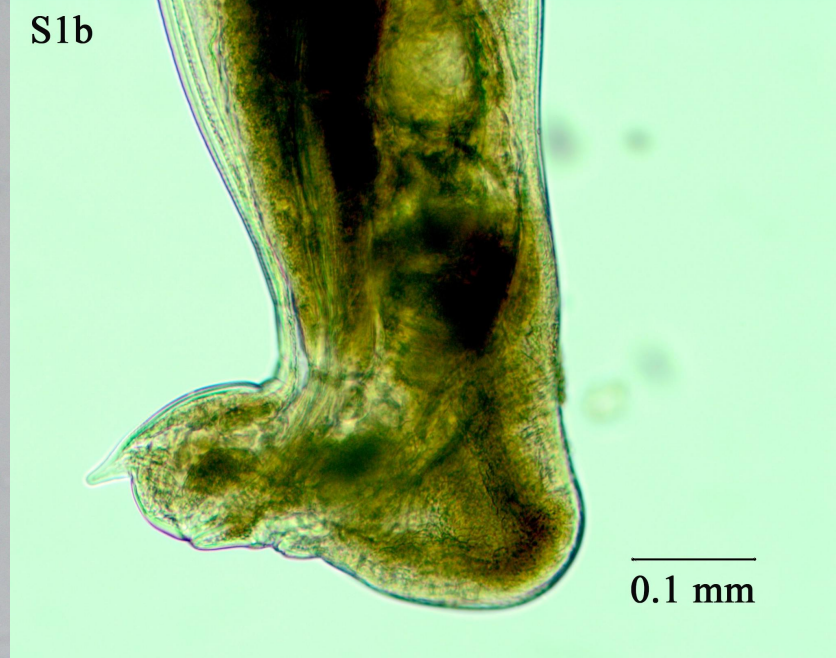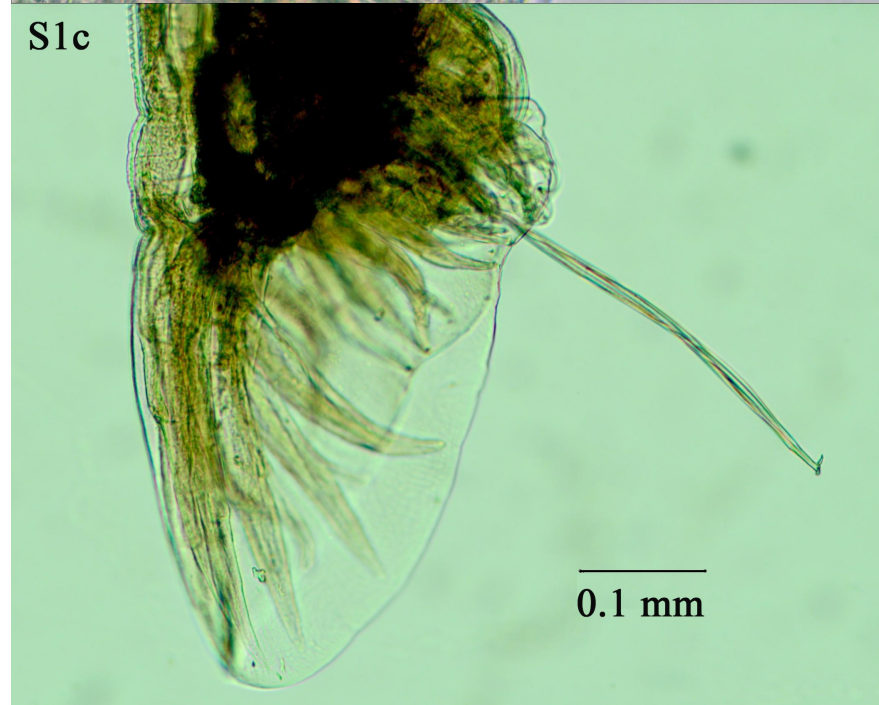

Supplement: Figure S1 — The detail information of morphological characteristics of Cyathostomum catinatum. (A) The head of Cy. catinatum, (B) The tail of female, (C) The tail of male, Bar = 0.1 mm. [file Image1.PDF]

S2a

0.1 mm

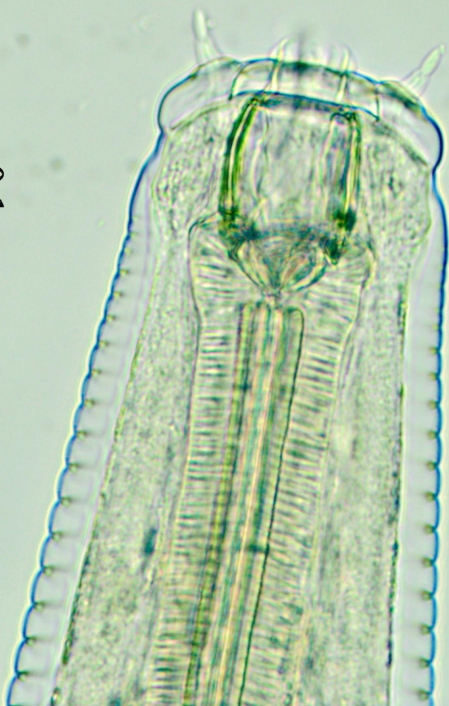

S2b

0.1 mm

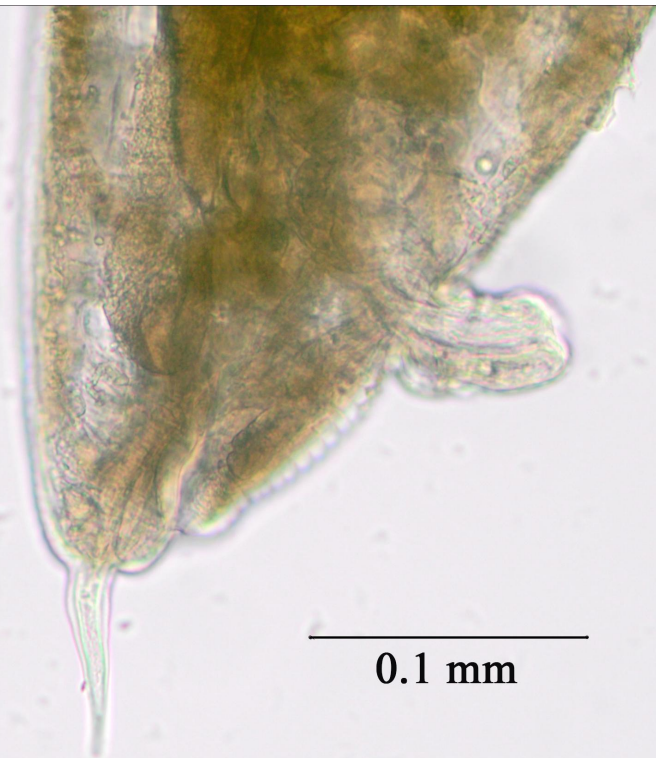

S2c

0.1 mm

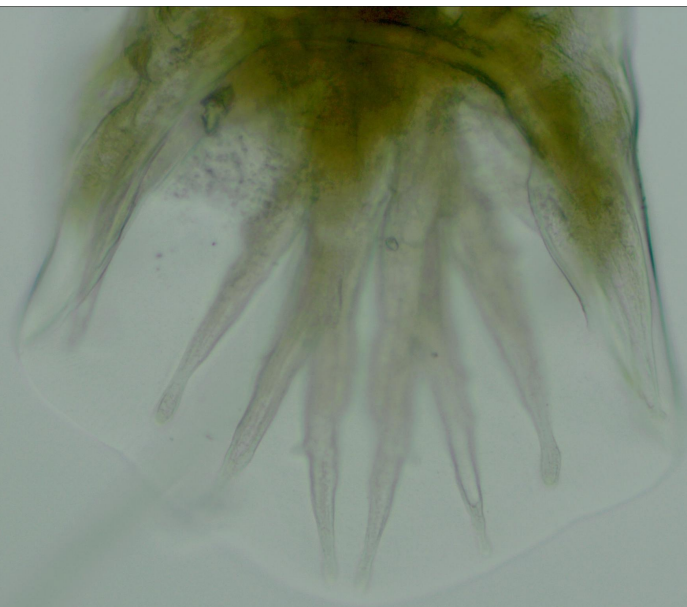

Supplement: Figure S2 — The detail information of morphological characteristics of Cylicostephanus minutus. (A) The head of Cs. minutus, (B) The tail of female, (C) The tail of male, Bar = 0.1 mm. [file Image2.PDF]

S3a

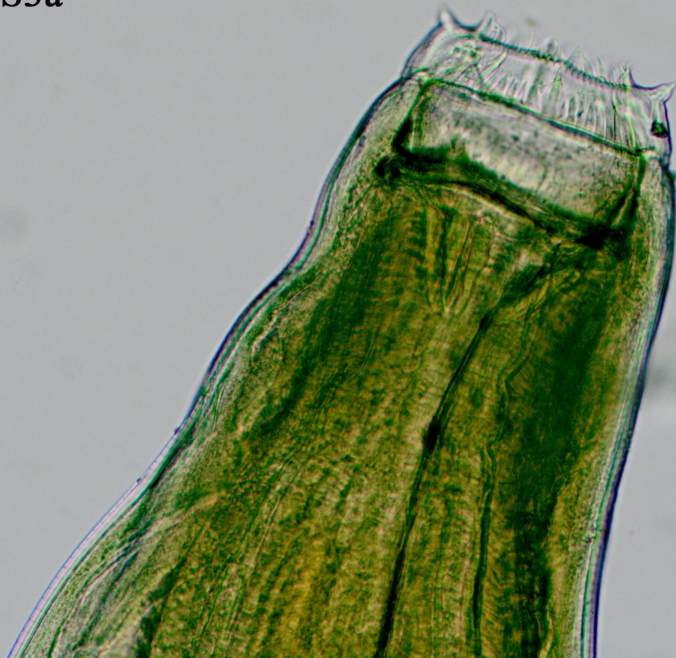

0.1 mm

S3b

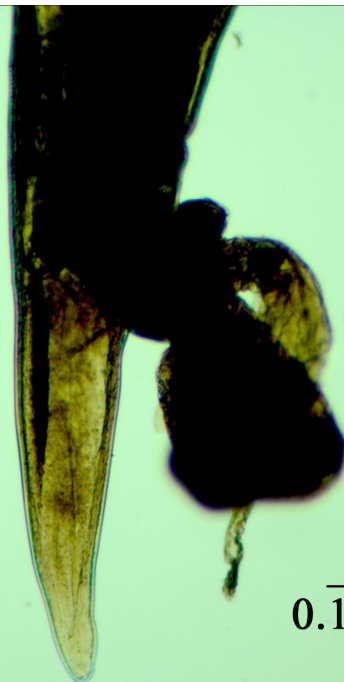

0.1 mm

S3c

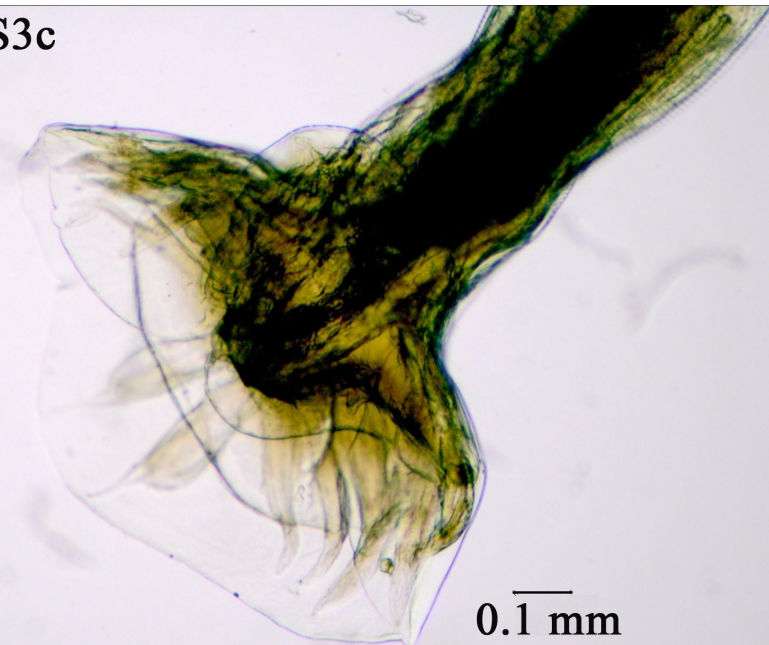

0.1 mm

Supplement: Figure S3 — The detail information of morphological characteristics of Poteriostomum imparidentatum. (A) The head of P. imparidentatum, (B) The tail of female, (C) The tail of male, Bar = 0.1 mm. [file Image3.PDF]
